# Diagnosis and follow-up of treatment of latent tuberculosis; the utility of the QuantiFERON-TB Gold In-tube assay in outpatients from a tuberculosis low-endemic country

**DOI:** 10.1186/1471-2334-10-57

**Published:** 2010-03-08

**Authors:** Anne M Dyrhol-Riise, Gerd Gran, Tore Wentzel-Larsen, Bjørn Blomberg, Christel Gill Haanshuus, Odd Mørkve

**Affiliations:** 1 Department of Medicine, Section of Infectious diseases, Haukeland University Hospital, Bergen, N-5021, Norway; 2 Department of Pulmonary Medicine, Haukeland University Hospital, N-5021 Bergen, Norway; 3 Centre for Clinical Research, Haukeland University Hospital, N-5021 Bergen, Norway; 4 Institute of Medicine, The Medical Faculty, University of Bergen, Bergen, N-5021 Norway; 5 Centre for International Health, University of Bergen, Bergen, N-5021 Norway

## Abstract

**Background:**

Interferon-gamma (IFN-γ) Release Assays (IGRA) are more specific than the tuberculosis skin test (TST) in the diagnosis of latent tuberculosis (TB) infection (LTBI). We present the performance of the QuantiFERON^®^-TB Gold In-tube (QFT-TB) assay as diagnostic test and during follow-up of preventive TB therapy in outpatients from a TB low-endemic country.

**Methods:**

481 persons with suspected TB infection were tested with QFT-TB. Thoracic X-ray and sputum samples were performed and a questionnaire concerning risk factors for TB was filled. Three months of isoniazid and rifampicin were given to patients with LTBI and QFT-TB tests were performed after three and 15 months.

**Results:**

The QFT-TB test was positive in 30.8% (148/481) of the total, in 66.9% (111/166) of persons with origin from a TB endemic country, in 71.4% (20/28) previously treated for TB and in 100% (15/15) of those diagnosed with active TB with no inconclusive results. The QFT-TB test was more frequently positive in those with TST ≥ 15 mm (47.5%) compared to TST 11-14 mm (21.3%) and TST 6-10 mm (10.5%), (p < 0.001). Origin from a TB endemic country (OR 6.82, 95% CI 1.73-26.82), recent stay in a TB endemic country (OR 1.32, 95% CI 1.09-1.59), duration of TB exposure (OR 1.59, 95% CI 1.14-2.22) and previous TB disease (OR 11.60, 95% CI 2.02-66.73) were all independently associated with a positive QFT-TB test. After preventive therapy, 35/40 (87.5%) and 22/26 (84.6%) were still QFT-TB positive after three and 15 months, respectively. IFN-γ responses were comparable at start (mean 6.13 IU/ml ± SD 3.99) and after three months (mean 5.65 IU/ml ± SD 3.66) and 15 months (mean 5.65 IU/ml ± SD 4.14), (p > 0.05).

**Conclusion:**

Only one third of those with suspected TB infection had a positive QFT-TB test. Recent immigration from TB endemic countries and long duration of exposure are risk factors for a positive QFT-TB test and these groups should be targeted through screening. Since most patients remained QFT-TB positive after therapy, the test should not be used to monitor the effect of preventive therapy. Prospective studies are needed in order to determine the usefulness of IGRA tests during therapy.

## Background

The Interferon-gamma (IFN-γ) Release Assays (IGRA), offering better specificity in the diagnosis of latent tuberculosis (TB) infection (LTBI) than the tuberculosis skin test (TST) [1-6], are now recommended in many national TB programs in low-endemic countries [6-8]. There are two commercial assays available and although T-SPOT.TB^® ^seems to give higher sensitivities in immunocompromised patients [9], the QuantiFERON^®^-TB Gold In-tube (QFT-TB) is often the test of choice in the clinical setting due to easier logistics when processing samples. In a meta-analysis the pooled sensitivity for QFT-TB was 70-78% and the specificity was 99% among non-BCG-vaccinated and 96% among BCG-vaccinated persons. The authors conclude that the IGRAs have excellent specificity that is unaffected by BCG vaccination [6].

Many studies have focused on the performance of the IGRA tests in active TB [4] or in certain risk groups as TB contacts [3,5], health-care workers [10] or in patients treated with tumor necrosis factor-alfa (TNF-α) inhibitors [11,12]. Fewer studies have been performed in outpatient clinical settings including individuals referred for various reasons according to clinical practise and national guidelines [9]. All studies are limited by the lack of a diagnostic gold standard for LTBI. The effect of preventive therapy on IFN-γ responses [13-16] and the cost-effectiveness of the IGRA tests on this patient population are still controversial [17,18]. Diel *et al*. reported that QFT-TB is a more accurate indicator of progression to active TB than TST [19]. Still, there is limited data concerning the usefulness of the IGRA tests to identify those individuals with LTBI who are at most risk for developing active disease and therefore most likely to benefit from preventive therapy [20].

Norway is a TB low-endemic country and the Norwegian population has until 2009 been BCG vaccinated at the age of fourteen, whereas the immigrant groups are often vaccinated as infants. Further, non-tuberculous mycobacteria (NTM) infections are also quite common [21,22]. These factors cause difficulties in diagnosing LTBI since the specificity of the TST test is low and variable in the BCG-vaccinated population. Immigration from TB high-endemic countries and increased global travelling with possible TB exposure challenge the epidemic situation [23]. Thus, the various groups demonstrating a positive TST test are very heterogeneous and more reliable diagnostic tools are needed to identify those with LTBI in order to offer proper preventive therapy and follow-up.

We performed a study to evaluate the usefulness of the QFT-TB test in the diagnosis of active and latent TB in a typical outpatient clinic in a TB low-endemic country. We analysed the various risk factors for a positive QFT-TB test. Further, we studied the reversion rates of the QFT-TB test and the IFN-γ responses right after and one year after ended preventive TB therapy in QFT-TB positive individuals.

## Methods

### Study participants

Persons referred to the TB outpatient clinic at Haukeland University Hospital, Bergen, Norway in the period January 2006 to January 2007 were included in the study. The participants were referred for medical evaluation of latent or active TB based on a positive TST and/or suspected exposure of TB according to national guidelines for screening [24]. The TST was performed in the primary health care institutions with purified protein derivative (PPD) RT 23 (2 TU) (Statens Serum Institute, Copenhagen, Denmark) and read after 72 hours. A cut-off value of ≥ 6 mm induration was considered a positive test. According to national guidelines, persons with TST reactions in the range of 6-14 mm should be referred if any risk factor for TB is known. Everyone demonstrating a TST of ≥ 15 mm should be evaluated by a specialist regardless of any known risk factor for TB. There was a delay from TST testing to specialist evaluation when active TB was not suspected. Thus, most of the TST were performed at least three months prior to inclusion. Thoracic X-ray and clinical examination were performed and an induced sputum sample was obtained for acid fast staining and culture in all participants according to standard procedures at the hospital.

When informed consent was given to participate in the study, a questionnaire translated to the native language regarding demographics, previous BCG vaccination and TST results, risk of exposure to TB and travel history was filled. The BCG vaccine status was established by self-reporting and the observation of a BCG scar by the specialist. The participants were divided in three groups: 1) from non-TB endemic country, 2) from non-TB endemic country/visited a TB endemic country and 3) from TB endemic country. The youngest participant was nine years old. Thus, age was categorised into six groups (9-19 years, 20-29 years, 30-39 years, 40-49 years, 50-59 years and ≥ 60 years). Duration of exposure was categorised into six groups (< 1 day, 1 to 6 days, 1 to < 4 weeks, 1 to < 2 months, 2 to < 3 months, ≥ 3 months). Time since last stay in a TB endemic country, time since exposure of TB and time since vaccination were all categorised as < 1 year, 1 to < 2 years, 2 to < 3 years, 3 to < 5 years, 5 to < 10 years, 10 to < 20 years and then in 10 year intervals. Preventive therapy with isoniazid and rifampicin for three months were started based on national guidelines [24]. The patients were followed with repetitive QFT-TB tests after three months and 15 months. Routinely HIV-test was not performed, but one of the participants was known HIV positive.

The study was approved by the Regional Ethics Committee for Medical Research and permission was given from the Norwegian Data Inspectorate. Written informed consent was given before inclusion in the study.

### QuantiFERON-TB Gold In-tube assay

One ml of whole blood was added to each of the three QFT-TB tubes; TB antigen (ESAT-6, CFP-10 and TB 7.7), mitogen positive control (phytohemagglutinin [PHA]) and a negative control. The tubes were treated as recommended by the manufacturer (Cellestis Ltd., Victoria, Australia) and the IFN-γ concentrations (IU/mL) in plasma was measured by an ELISA reader and calculated by the 'QFT-TB-analysis Software'. An IFN-γ ≥ 0.35 IU/ml (TB antigens minus negative control) was considered a positive test. A determinate test must have mitogen minus negative control ≥ 0.5 IU/ml and/or TB antigens minus negative control ≥ 0.35 IU/ml. Values > 10 IU/ml was treated as 10 IU/ml due to inaccurately of the ELISA assay above this level.

### Statistics

Statistical analysis was performed using Stata 11 (Stata Corporation, College Station, Texas, US). Data are presented as mean values with range and standard deviation (SD). Univariate assessment of risk factors for positive QFT-TB test was done by Chi square test and t test as appropriate. Univariate odds ratios (OR) were obtained by applying logistic regression on each variable separately and presented with 95% confidence intervals (CI).

Multivariate analysis of risk factors for positive QFT-TB test was performed by logistic regression. All variables in the univariate analysis were included in the multivariate model. Since observations with missing values are automatically removed from logistic regression models, only 389 of the total of 481 observations remained in the final multivariate model. The observation "duration of exposure" had a particularly high number of missing values (n = 63) and was responsible for a large part of the missing values in the final model. We performed the logistic regression on the final model with and without that variable, and found that excluding or including it did not significantly alter the findings in the model.

A priori, we considered the most important potential interactions in the model to be those between BCG vaccine status and other explanatory variables. We assessed all these potential interactions and did not find any significant interactions between BCG vaccine status and any of the other explanatory variables. Trends of QFT-TB values over time were assessed by Wilcoxon rank-sum test.

## Results

### Epidemiological and clinical data

A total of 481 persons referred to the TB outpatient clinic for examination of possible TB infection were included in the study (Table 1). Fifteen patients were diagnosed with new active TB and 444 were suspected of latent TB defined by a positive TST ≥ 6 mm. Fifty-four had thoracic X-ray findings that needed evaluation for possible active TB. This was typically found in the groups of previous TB disease (7/8) or with origin from a TB endemic country (18/86). Induced sputum was obtained from all the participants and twelve had positive culture of *M. tuberculosis *and one of *M. fortuitum*. Two additional patients were regarded as active TB disease based on X-ray findings and one patient was diagnosed with glandular TB. Four of the patients with active TB, two of them with positive TB culture, demonstrated normal X-rays. Among the 15 patients with active TB, 13 was from a TB endemic country (mean 5.2 years since arrival Norway, range 0-31, SD ± 8.1), one had parents from a TB endemic country, but was born in Norway and one was ethnical Norwegian.

**Table 1:** Indications for Tuberculin skin test screening and/or referral to clinical specialist evaluation (n = 481)

Screening	No	%
Contact Investigation*^)^	264	55.1
Immigrants*^)^	86	17.9
Health-care workers**^)^	7	1.5
School children before BCG vaccination	27	5.6
Before treatment with TNF-α inhibitor*^)^	64	13.3
HIV-1 infection	1	0.2

**Clinical evaluation**		

Thoracic X-ray findings (only)	19	3.9
Previous TB disease (only)	8	1.7
Suspected extrapulmonary TB	5	1.0

TNF-α = tumor necrosis factor-alfa.

*) Including persons with previous TB and/or X-ray findings.

**) Working ≥ 3 months in a TB endemic country.

In a total of 435 (90.4%) persons a TST was performed before referral, whereas in 46 (9.6%) persons no TST was obtained, mainly because of documented previously strong response. The mean TST value for the total group was 13.3 mm (range 0-30). All patients with active TB had a positive TST (mean 18 mm, range 10-24 mm).

The epidemiological data are as described in Table 2. A total of 166 persons (34.5%) were born in a non-western TB high (≥ 100/100 000) or intermediate (10-100/100 000) prevalence country, the majority from sub-Saharan Africa or Southeast Asia. The mean time since immigration to Norway was 5.5 years (range 0-31, SD ± 6.7). A total of 309 (64.0%) of the participants reported stay in a TB endemic country, but only 208 (44.7%) stayed in a period of 3 months or more and of these only 38 were ethnically Norwegians. The mean time since the last visit was 5 years (range 0-47, SD ± 7.3).

**Table 2:** Characteristics of study participants (n = 481)

Characteristics	No	%
Age; mean (range)	39 (9-87)	
Gender		
Male	196	40.7
Female	285	59.3
Origin		
Norway with western parents	296	61.5
Another western country	7	1.5
Parents from TB endemic country	12	2.5
TB endemic country	166	34.5
Stay in TB endemic country	309	64.0
Exposure of TB	261	54.6
BCG vaccinated	423	87.9
BCG scar	356	74.0
Previous TB	28	5.8

Altogether 261 (54.6%) of the participants reported that they had been exposed to a known case of TB, the majority referred as part of a contact investigation. However, the majority of persons from TB endemic countries and seven of the 15 patients diagnosed with active TB during the study did not report on known exposure. The mode of contact between the TB case and the study participants was as shown in Table 3. The mean time period since exposure was 3.8 years (range 0-61, SD ± 10.5).

**Table 3:** Contact investigations: exposure of tuberculosis and QuantiFERON-TB Gold results compared with no exposure.

TB case	Exposure No (% of total)	Pos QFT-TB (% of risk group)
Household	42 (8.7)	59.5
Family outside household	41 (8.5)	19.5
Work	104 (21.6)	15.5
Friends/occupational	59 (12.3)	39.0
School	10 (2.1)	40.0
Aeroplane	5 (1.0)	0
No reported exposure	220 (45.7)	32.3

A total of 423 (87.9%) persons reported that they had received BCG vaccination, but scar was only found in 356 (74%). The mean time since vaccination was 27.7 years (range 1-64, SD ± 13.4), varying according to the country of origin, since most of the foreign persons were vaccinated as infants while BCG vaccination in Norway is performed at the age of fourteen.

Twenty-eight (5.8%) of the participant had previously experienced active TB disease and 26 reported that they had received treatment from 3-24 months. Thirteen of the treated were born in Norway or another western country and thirteen came from TB endemic countries. The mean time period since TB treatment was 10.3 years (range 1-22, SD ± 7.0) and 35.7 years (range 1-79, SD ± 27.3) for the immigrants and ethnical Norwegians, respectively.

### QuantiFERON-TB Gold and Tuberculin skin test results

**Figure 1:**
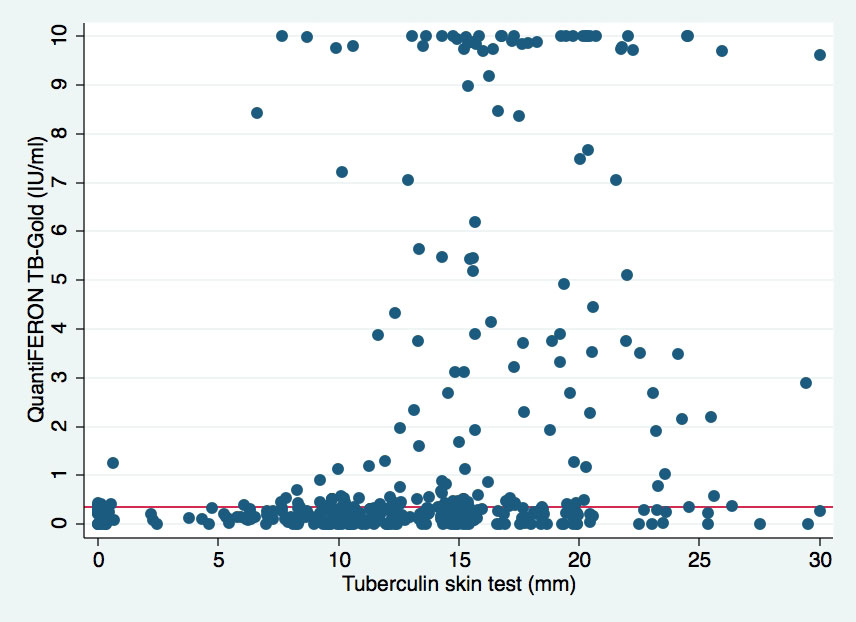
**Relationship between Tuberculin skin test and QuantiFERON-TB Gold responses**. Corresponding tuberculin skin test (mm) and QuantiFERON-TB Gold (QFT-TB, UI/ml) responses at inclusion in patients with suspected tuberculous infection; positive QFT-TB test (n = 122) and negative QFT-TB test (n = 308). The cut-off for positive test is ≥ 0.35 IU/ml (solid line). Values > 10 IU/ml are treated as 10 IU/ml due to inaccurately of the ELISA assay above this level.

The QFT-TB test was obtained at inclusion and the test was conclusive for all participants with no indeterminate results. All together 148 (30.8%) persons demonstrated a positive test. The correlation between TST and QFT-TB was as shown in Figure 1. The mean TST was 16.7 mm (range 0-30) for the QFT-TB positive group and 11.7 mm (range 0-30), (p = 0.018) for the QFT-TB negative group. The QFT-TB test was positive in all patients diagnosed with active TB, including the patient with *M. fortuitum*, but two patients had IFN-γ values right above cut-off, confirmed by repetitive testing (0.57-0.88 IU/ml).

The percentage of positive QFT-TB tests was highest in persons with TST ≥ 15 mm (47.5%) and this was significant compared to the groups with TST of 11-14 mm, 6-10 mm and < 6 mm (p < 0.001) (Table 4). The only person with a positive QFT-TB and TST < 6 mm had experienced active TB sixty years back, but had no findings to support reactivation of infection. The eleven with positive QFT-TB and TST ≤ 10 mm where either from TB endemic countries or ethnical Norwegians with known exposure, except one adolescent with a TST of 9 mm before BCG vaccination. A total of 87 (80.1%) of the 107 persons with a negative QFT-TB test and TST ≥ 15 mm were ethnical Norwegians.

**Table 4:** QuantiFERON-TB Gold (QFT-TB) results in the overall group of patients with a positive tuberculin skin test (≥ 6 mm) and in various TST subgroups

TST		Positive QFT-TB
**Induration (mm)**	**No (% of total)**	**No (% of TST group)**

≥ 6	398 (82.7)	127 (31.9)
0-5	37 (7.7)	1 (2.7)^2^
6-10	105 (21.8)	11 (10.5)
11-14	89 (18.5)	19 (21.3)
≥ 15	204 (42.4)	97 (47.5)^3^
No TST^1^	46 (9.6)	20 (43.5)

TST = tuberculin skin test. 1) Not tested due to previous strong response. 2) Previous tuberculosis, 3) p < 0.001 compared to the other TST groups.

The fraction of positive QFT-TB tests varied within the various TB risk groups (Additional file 1). The percentage of positive test was highest in persons born in a TB endemic country (111/166, 66.9%) or in those with previous TB disease (20/28, 71.4%). The fraction of positive QFT-TB test in those reporting exposure for TB was comparable with the study group in general. However, there was large variation within the various modes of exposures with the highest fraction of positive QFT-TB test found in the group exposed within the household (59.5%) and the lowest in the group exposed for TB at work (15.5%) (Table 3).

### Predictive factors for a positive QuantiFERON-TB Gold test

In the multivariate logistic regression analysis, origin from a TB endemic country (OR 6.82, 95% CI 1.73-26.82), duration of TB exposure (OR 1.59, 95% CI 1.14-2.22) and previous TB disease (OR 11.60, 95% CI 2.02-66.73) were all independently associated with a positive QFT-TB test (Additional file 1). Recent stay in a TB endemic country expressed as years since last stay was associated with a positive QFT-TB test (OR 1.32, 95% CI 1.09-1.59), while visit to a TB endemic country was not. The percentage of QFT-TB positive test was comparable in the groups with and without BCG vaccination. However, there still seems to be an association between BCG vaccination and a negative QFT-TB test (OR 0.13, 95% CI 0.03-0.66).

### QuantiFERON-TB Gold responses during preventive therapy for latent tuberculosis

Preventive therapy with isoniazid and rifampicin for three months was started in 57 persons with suspected LTBI. The decision to treat was made by the clinician and the QFT-TB test was known at the time of decision. Only one QFT-TB negative person was treated, a Norwegian women with TST > 30 mm, previous long-term stay in a TB endemic country and planned for therapy with a TNF-α inhibitor. The other 56 initiating therapy were all QFT-TB positive, representing 43% of the group with a positive QFT-TB test. The majority of the treated had origin from TB endemic countries, but 16 were Norwegians tested as part of a contact investigation. All of the treated, except four, had TST > 6 mm. The main reasons for not giving preventive therapy to QFT-TB positive individuals were older age, leaving the country, pregnancy or previous TB. Fifty persons received therapy for a total of three months. Seven stopped therapy, predominantly due to side effects of the drugs.

**Figure 2:**
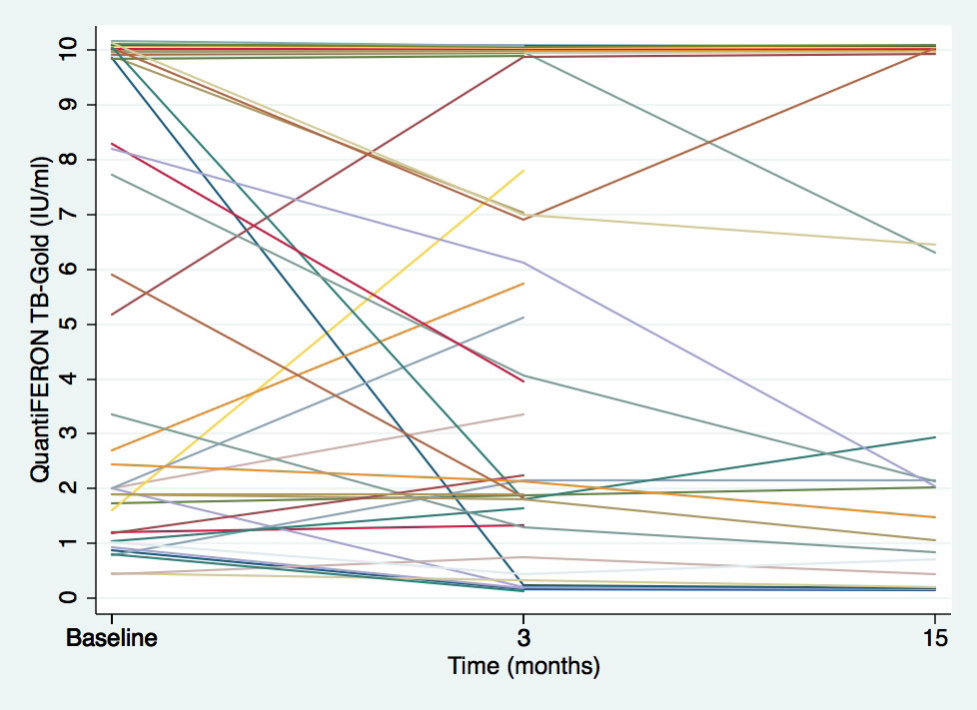
**QuantiFERON-TB Gold responses in latent tuberculosis during preventive therapy**. Interferon gamma responses (IU/ml) before (baseline, n = 44), after three months (at the end of therapy, n = 40) and after 15 months (one year after ended therapy, n = 26) in patients with latent tuberculosis treated with isoniazid and rifampicin.

Altogether 44 of the patients receiving preventive therapy were available for repetitive QFT-TB tests. After three months 87.5% were still QFT-TB positive (35/40 tested) whereas after 15 months, one year after the end of therapy, 84.6% remained positive (22/26 tested). All patients with reversed QFT-TB test after three months were still negative at 15 months. No patient reverted from positive to negative between three and 15 months although one additional person was negative at month 15, but no test was performed at three months. All negatives, except one, had low baseline IFN-γ responses (0.46-2.0 IU/ml). Compared to baseline, 44.7% showed a decline while 55.3% had similar or stronger IFN-γ responses after three months (Figure 2). The corresponding numbers after 15 months were 53.8% and 46.2%. By using an upper cut-off of the assay of 10 IU/ml, we found no significant difference in IFN-γ responses before (mean 6.13 IU/ml ± SD 3.99) and after therapy (mean 5.65 IU/ml ± SD 3.66 vs. 5.65 IU/ml ± SD 4.14 after three and 15 months, respectively), (p > 0.05). Repetitive testing after three months of both QFT-TB positive and QFT-TB negative not receiving therapy showed that of the seven QFT-TB positive (0.40-10 IU/ml) tested, five were still positive (0.41-10 IU/ml). All seven QFT-TB negative were still negative.

## Discussion

We present a longitudinal study performed in a regular outpatient TB clinic in a TB low-endemic country. In contrast to a number of studies focusing on particular populations [2,4,5,11,25,26], we investigated the performance of the QFT-TB test in various risk populations referred according to present national guidelines [24] and thus reflecting the clinical situation at a large size Norwegian hospital. We further present data of IFN-γ responses during preventive therapy of latent TB.

### Risk factors for a positive QuantiFERON-TB Gold test

We show that the majority of the persons referred for evaluation of TB based on a positive TST were QFT-TB negative. If the diagnosis is based on a positive QFT-TB test only 30% of the patients studied have TB infection. We found that in a TB low-endemic country a positive QFT-TB test is associated with origin from or short time since stay in a TB endemic country. We observed that although many ethnical Norwegians reported having visited a TB endemic country, very few stayed for longer periods and the majority of these were QFT-TB negative. Our data support other studies, including the Norwegian study of asylum seekers [25,27], and underscores the importance of screening asylum seekers for TB at immigration.

Duration of TB exposure rather than exposure or not was associated with a positive QFT-TB-test. Thus, we found a very low fraction of positive QFT-TB tests in the group exposed for TB at work in contrast to exposure within the household. In our hospital setting exposure-time seems to be short and low-risk due to proper action during high-risk procedures [28]. Our findings are partly in contrast to Vinton *et al.'s *study where high-risk exposure, but not duration of exposure, was associated with a positive test [25].

We found that previous TB diagnosis was also associated with a positive QFT-TB test, which contrasts findings by Winje *et al*. [27]. Over 70% of those with previous TB still harboured a positive QFT-TB test although anti-tuberculous therapy had been given to the majority several years earlier. IGRA tests, based on short time *in vitro *stimulation, are supposed to predominantly measure effector T cells as indication of persistence of TB antigens [29]. Still, the test does not allow discriminating between presence of latent or subclinical infection and TB specific immunological memory. Therefore, our data do not support that IGRA-tests can be used to discriminate between various stages of TB infection or response to therapy. Finally, we found an association between QFT-TB negative test and BCG vaccination. This could be explained by the fact that almost 100% of the ethnical Norwegians with low percentage of positive QFT-TB test were vaccinated. An alternative explanation is that vaccination in infancy protects against TB infection [30].

In conclusion, recent immigrants from TB endemic countries and those with long duration of exposure seem to be most at risk for TB infection defined by a positive QFT-TB test. Based on our calculations (data not shown) a two-step approach seems to be most cost-effective when screening for LTBI compared to the previous strategy using TST alone. This is in agreement with other studies [17,18,31], and our data has been used in the revised national guidelines [7].

### QuantiFERON-TB Gold and Tuberculin skin test responses

The number of QFT-TB positive tests increased with increasing TST values, although we have not performed a direct comparison between TST and QFT-TB, since a positive TST was one of the major criteria for referring the patients. Only 2.7% (one with previous active TB) and 10.5% had a positive QFT-TB test in individuals with TST < 6 mm and < 11 mm, respectively. This is comparable with earlier studies of contact investigations including the Norwegian study of asylum seekers [27,32,33]. Our data also supports a German study that found that TST-/QFT+ discordance increased with age [34], and indicate that the QFT-TB test might be more sensitive than TST in the elderly.

TST ≥ 15 mm has previously been considered true TB infection [21,35] and has been shown to correlate to progression of active disease [36,37]. However, only 47.5% of the individuals with such strong TST responses had a positive QFT-TB test. There is no certain explanation for this discordance [38-41]. The clinical effect of NTMs on TST is normally minimal, except in parts of the world where TB prevalence is low and NTMs more common due to moist climate [21]. It is of interest that of those 107 persons with TST ≥ 15 mm and negative QFT-TB tests only twenty were from TB endemic countries. Since very small effects of BCG on TST reactions are expected after 10 years for people vaccinated in infancy [21], this might represent false negative QFT-TB tests [6]. Still, our data indicate that BCG vaccination, boosting of immune responses by repetitive testing [42] or infections with NTMs [21] are responsible for the vast majority of false positive TST. Nevertheless, lack of diagnostic gold standard for LTBI emphasises the need for follow-up of individuals with strong TST reactions and risk factors for TB infection.

### QuantiFERON-TB Gold responses during preventive therapy for latent tuberculosis

The effect of standard three months preventive TB treatment on QFT-TB was only modest with 85% still positive both shortly after and one year after ended therapy. The few cases with negative conversion had predominantly low values at baseline. In contrast, cases with strong responses ≥ 10 IU/ml more often persisted. The prognostic use of IGRA tests as marker for response to therapy is not established and data are conflicting, performed in various endemic settings and with different IGRAs and upper and lower cut-off levels. Most studies have been performed on patients treated for active TB [43-52]. Pai *et al*. show persistence of QFT-TB responses during treatment [48]. In contrast, Katiyar *et al*. found that only 48% were positive by the same assay after 6 months [51]. Also studies of T-SPOT.TB are conflicting as Dheda *et al*. report 81% with negative test in late phase therapy of patients with active TB [49]. The corresponding numbers in Ribeiro *et al's *study is only 10% [52]. Studies of LTBI are limited and data are conflicting also in this group lacking the diagnostic gold standard for comparison [13-16]. Our study is performed in a low transmission setting with little likelihood of reinfection. Further, the patients were also treated in a DOTs regimen ensuring a good compliance to therapy. Combined this indicates that persistent positive QFT-TB tests represent persistence of pre-treatment IFN-γ responses.

Around half of the patients experienced a decline, while the other half maintained strong IFN-γ responses. However, all patients were still healthy 15 months after preventive therapy with no signs of active TB. Although it has been proposed that IGRA tests could identify those who will progress to active TB [53], in the study by Diel *et al*. only the minority of the QFT-TB positive individuals recently exposed developed active disease during the two years follow-up [19].

The interpretation of serial testing of IGRA tests is controversial. For the baseline QFT-TB we performed dual testing of the same sample with almost 100% reproducibility (data not shown). Unfortunately we were not able to repeat systematically the QFT-TB test for all positive not treated or for the QFT-TB negative group, but our limited data indicate an overall good intra-assay reproducibility over time although two converted from low-level positive values to negative. Still, low-level positive QFT-TB tests need to be interpreted with caution as transient responses to QFT-TB are quite common [54] and positive results can vary over time [55]. Thus, we can not for sure conclude that the reversion of QFT-TB from positive to negative were due to the effect of therapy since some of the cases had low baseline values. Finally, although TST originally has been considered not to boost IGRA responses, data has been conflicting [56,57]. A recent report demonstrates that this could occur up to three months after TST [58]. In our study TST testing in the primary health care system was performed in the majority of cases at least three months prior to the QFT-TB testing minimising this effect. Still, small variations around the cut-off values should be treated with caution when deciding for LTBI therapy and new cut-offs might be validated.

In conclusion, our data indicate that IGRA tests should not be used for the individual as a reliable marker to monitor the effect of therapy.

### Performance of the QuantiFERON-TB Gold test in an outpatient clinical setting

The performance of the QFT-TB test was good in this clinical setting. All 15 diagnosed with active TB had a positive QFT-TB test giving a sensitivity of 100% and a high agreement with TST. The sensitivities of both TST and IGRA tests in larger studies of active TB are in the range of 75-90% depending of the assay [6]. Although a much too small number to conclude, our data suggest that patients with active TB diagnosed in an outpatient clinical setting might possess a less advanced stage of disease with preserved reactivity to TB antigens.

We got no indeterminate results although more than 13% of the participants were tested before treatment with a TNF-α inhibitor and assumed to have suppressed immune systems to various extents. We have in our diagnostic laboratory later experienced approximately 10% indeterminate results in immunocompromised patients (unpublished data). The only HIV positive included had a rather high CD4 count and tested positive with the QFT-TB test. HIV testing was decided by the clinician and has traditionally not been performed in our TB outpatient clinic. However, the lack of routinely HIV testing is a limitation in our study as it is well known that IGRA tests could be inconclusive in immunocompromised patients, especially with CD4 count under 100 [9,26]. Although the T-SPOT.TB assay appears to give higher sensitivity and rates of conclusive tests than the QFT-TB test [6], one must be aware of the limitations of both IGRA tests in patients with suppressed immune system.

## Conclusion

The number of TB infected based on a positive QFT-TB test seems to be only one third of that based on a positive TST. Recent stay or origin from TB endemic countries and long duration of exposure seems to be risk factors for LTBI and thus these groups should be targeted through screening. Most patients with LTBI were still QFT-TB positive after preventive therapy and the test should not be used as a reliable marker to monitor the effect of therapy. Prospective studies are needed in order to understand the kinetic of IFN-γ responses and determine the usefulness of IGRA tests as diagnostic tool during therapy.

## Competing interests

The authors declare that they have no competing interests.

## Authors' contributions

**AMDR **has initiated and designed the study, participated in interpretation of data and statistical analysis and drafted the manuscript. **GG**: has participated in recruiting study participants and revising of the manuscript. **TWL**: has participating in statistical analysis and revising of the manuscript. **BB**: has participating in statistical analysis, interpretation of data and revising of the manuscript. **CGH**: has participated in experimental work and revising of the manuscript. **OM**: has participated in recruiting study participants and revising of the manuscript. All authors have read and approved the final manuscript.

## Pre-publication history

The pre-publication history for this paper can be accessed here:

http://www.biomedcentral.com/1471-2334/10/57/prepub

## Supplementary Material

Additional file 1 **Predictors for positive QuantiFERON-TB Gold test (QFT-TB)**. Multivariate analysis performed by logistic regression. * P-value < 0.05, **P-value < 0.001. CI: confidence intervals, OR: odd ratios. Age was categorised as 9-19 years, 20-29 years, 30-39 years, 40-49 years, 50-59 years and ≥ 60 years. Recent stay in a TB endemic country, recent exposure of TB and recent vaccination were all categorised as time since last event (<1 year, 1 to <2 years, 2 to <3 years, 3 to <5 years, 5 to <10 years, 10 to <20 years and then in 10 year intervals). Duration of exposure was categorised as <1 day, 1 to 6 days, 1 to <4 weeks, 1 to <2 months, 2 to <3 months, ≥ 3 months. Some numbers do not add up to 481 for all characteristics due to unknown parameters for some participants. The number of observations available for each variable and the total number of observations (no = 389) included in the final logistic regression model are as given in brackets. Click here for file
