# Supplementary material for: Diagnosis and follow-up of treatment of latent tuberculosis; the utility of the QuantiFERON-TB Gold In-tube assay in outpatients from a tuberculosis low-endemic country
Source: BMC Infect Dis. 2010 Mar 8;10:57. doi: 10.1186/1471-2334-10-57 (PMC2842274; doi:10.1186/1471-2334-10-57)
Supplement: Additional file 1 — Predictors for positive QuantiFERON-TB Gold test (QFT-TB). Multivariate analysis performed by logistic regression. * P-value < 0.05, **P-value < 0.001. CI: confidence intervals, OR: odd ratios. Age was categorised as 9-19 years, 20-29 years, 30-39 years, 40-49 years, 50-59 years and ≥ 60 years. Recent stay in a TB endemic country, recent exposure of TB and recent vaccination were all categorised as time since last event (<1 year, 1 to <2 years, 2 to <3 years, 3 to <5 years, 5 to <10 years, 10 to <20 years and then in 10 year intervals). Duration of exposure was categorised as <1 day, 1 to 6 days, 1 to <4 weeks, 1 to <2 months, 2 to <3 months, ≥ 3 months. Some numbers do not add up to 481 for all characteristics due to unknown parameters for some participants. The number of observations available for each variable and the total number of observations (no = 389) included in the final logistic regression model are as given in brackets. [file 1471-2334-10-57-S1.DOC]

**Table 5**. Predictors for positive QuantiFERON-TB Gold test (QFT-TB)

|  |  | **Univariate (n=481)** | **Multivariate (n=389)** |
| --- | --- | --- | --- |
| **Characteristics** | **Pos QFT-TB % (n/N)** | **OR (95% CI)** | **OR (95% CI)** |
| Sex (N=481) |  |  |  |
| Female | 30.6 (60/196) | 1 | 1 |
| Male | 30.9 (88/285) | 1.01 (0.68-1.50) | 0.90 (0.49-1.65) |
| Increasing age (N=481) |  | 0.88 (0.78-1.00) | 1.24 (0.89-1.71) |
| Stay in TB endemic country (N=481) |  |  |  |
| Never | 14.0 (24/172) | 1 | 1 |
| Visited TB endemic country | 9.1 (13/143) | 0.62 (0.30-1.26) | 0.13 (0.03-0.65) |
| Origin from TB endemic country | 66.9 (111/166) | 12.45 (7.26-21.33)** | 6.82 (1.73-26.82)* |
| Recent stay (N=474) |  | 1.22 (1.15-1.30)** | 1.32 (1.09-1.59)* |
| Exposure to TB (N=480) |  |  |  |
| No | 32.6 (71/218) | 1 | 1 |
| Yes | 29.4 (77/262) | 0.86 (0.58-1.27) | 0.33 (0.02-4.94) |
| Duration of exposure (N=418) |  | 1.04 (0.96-1.13) | 1.59 (1.14-2.22)* |
| Recent exposure (N=468) |  | 0.97 (0.93-1.02) | 0.99 (0.74-1.33) |
| BCG vaccination (N=459) |  |  |  |
| No | 30.6 (11/36) | 1 | 1 |
| Yes | 29.6 (125/423) | 0.95 (0.46-2.00) | 0.13 (0.03-0.66)* |
| Recent vaccination (N=465) |  | 0.99 (0.86-1.13) | 1.09 (0.77-1.55) |
| Previous TB (N=481) |  |  |  |
| No | 28.3 (128/453) | 1 | 1 |
| Yes | 71.4 (20/28) | 6.35 (2.73-14.78)** | 11.60 (2.02-66.73)* |

Multivariate analysis performed by logistic regression. * P-value <0.05, **P-value<0.001. CI: confidence intervals, OR: odd ratios. Age was categorised as 9-19 years, 20-29 years, 30-39 years, 40-49 years, 50-59 years and ≥ 60 years. Recent stay in a TB endemic country, recent exposure of TB and recent vaccination were all categorised as time since last event (<1 year, 1 to <2 years, 2 to <3 years, 3 to <5 years, 5 to <10 years, 10 to <20 years and then in 10 year intervals). Duration of exposure was categorised as <1 day, 1 to 6 days, 1 to <4 weeks, 1 to <2 months, 2 to <3 months, ≥ 3 months. Some numbers do not add up to 481 for all characteristics due to unknown parameters for some participants. The number of observations available for each variable and the total number of observations (no=389) included in the final logistic regression model are as given in brackets.
